# In Search of Ultra-Black Ceramic Pigments Using Microwaves: Delafossite Cuprates CuMO_2_ (M = Mn, Fe, Cr)

**DOI:** 10.3390/ma18214910

**Published:** 2025-10-27

**Authors:** Guillermo Monrós, Vicente Esteve, Carolina Delgado, Guillem Monrós-Andreu, Mario Llusar

**Affiliations:** 1 Department of Inorganic and Organic Chemistry, University Jaume I, 12071 Castelló de la Plana, Castelló, Spain; estevev@uji.es (V.E.); caroladelg@gmail.com (C.D.); mllusar@uji.es (M.L.); 2 Department of Mechanic Engineering and Construction, University Jaume I, 12071 Castelló de la Plana, Castelló, Spain; gmonros@uji.es

**Keywords:** ceramic pigment, delafossite, microwaves, blackness, ultra-black, ceramic glaze

## Abstract

Cuprate delafossite phases such as CuMnO_2_ (crednerite) and CuFeO_2_, as well as iron- and manganese-doped mcconnellite composites, were investigated as candidates for producing intense black ceramic pigments via conventional solid-state synthesis. Both electric kiln and fast dielectric (microwave) firing methods were employed, with mcconnellite (CuCrO_2_) used as a reference pigment. Microwave firing led to a marked improvement in sample blackness compared to conventional electric firing. Among the delafossite phases, only mcconnellite subjected to microwave-assisted firing (R_Vis_ = 1.40%, corresponding to 98.60% visible light absorption) emerges, pending further optimization, as a promising candidate for an ultra-black ceramic pigment (R_Vis_ < 1%) under optimized glaze conditions (ZnO-free) and a firing temperature of 1000 °C. Considering the pigments in powder form, microwave-fired crednerite (R_Vis_ = 4.85%, 95.15% absorption) and iron- and iron–manganese-doped mcconnellite composites (R_Vis_ = 3.27% and 3.23%, respectively) appear as potential candidates for deep-black pigments (R_Vis_ < 3%), benefiting from the composite effect between the delafossite phase and the associated chromium spinel. Moreover, microwave-fired crednerite also demonstrates noteworthy potential for deep-black coloration in glazed samples (R_Vis_ = 4.27%, 95.73% absorption).

## 1. Introduction

Discovered by Friedel in 1873, the mineral CuFeO_2_ [1], named *delafossite* in honour of the French mineralogist and crystallographer Gabriel Delafosse, gave its name to the ABO_2_ oxide family (delafossites), which have recently attracted growing scientific interest [2,3,4], particularly since Kawazoe et al. demonstrated the coexistence of optical transparency and p-type conductivity in CuAlO_2_ [5,6]. Delafossite cuprates, with the general formula ABO_2_ (A^+^ = Cu; B^3+^ = Mn, Fe, Cr), possess a layered structure composed of BO_6_ octahedral sheets linked by linearly coordinated O–A^+^–O dumbbells. Pabst (1939) first described the trigonal delafossite structure (space group *R*-3*m*), while Köhler and Jansen later reported the equivalent hexagonal modification (space group *P*6_3_/*mmc*) [7,8]. At room temperature, however, crednerite (CuMnO_2_) adopts a unique monoclinic layered structure consisting of edge-shared MnO_6_ octahedra and linearly coordinated Cu^+^ ions. This arrangement is interpreted as a Jahn–Teller distortion of the delafossite structure induced by Mn^3+^ ions, making crednerite an intriguing derivative of the delafossite family with promising potential in catalytic applications [9].

The commercial sprayable coating Vantablack (a portmanteau of *VANTA*, “Vertically Aligned Nanotube Arrays,” and “black”) is an ultra-black surface (L*a*b*= 0.2/0.9/–1; visible reflectance R_Vis_ = 0.035%) produced by chemical vapour deposition (CVD). It absorbs up to 99.965% of incident visible light [10,11]. Ultra-black surfaces conceal surface irregularities and textures, eliminate glare, and render objects nearly invisible, making them highly valuable in space science and optical applications; for instance, in enhancing telescope sensitivity and suppressing stray light in precision optical instruments. In general, an ultra-black surface is defined as one that absorbs more than 99% of incident light (R_Vis_ < 1%) [10,11]. By contrast, commercial black pigments typically exhibit R_Vis_ values below 5%, while Industry Tint Reference Black (ITRB) coatings reach approximately R_Vis_ ≈ 3%. Therefore, a deep-black surface may be defined as one that absorbs more than 97% of visible light, corresponding to R_Vis_ < 3%.

In nature, several organisms exhibit ultrablack pigmentation as an adaptive trait shaped by selective pressures within their environments. This pigmentation enhances the visibility of sexually selected colours in peacock spiders and birds-of-paradise, provides thermoregulatory or camouflage advantages to vipers, and aids butterflies in evading predators. Such anti-reflective mechanisms based on ultrablack pigmentation play a crucial role in survival and reproductive success [12].

To produce ultrablack coloration, organisms have evolved sophisticated mechanisms centred on eumelanin (from the Greek *εὖ μέλας*, meaning “true black”). Eumelanin, the black component of the melanin pigment family, is synthesized through melanogenesis; a biochemical process involving the oxidation of the amino acid tyrosine to 5,6-dihydroxyindole, followed by polymerization [13]. However, while eumelanin can generate a deep black colour, it may still exhibit surface gloss, as seen in shiny fur or feathers. In contrast, ultrablack coloration lacks gloss and instead appears matte, owing to hierarchical surface microstructures that modulate light interactions and minimize reflection. Similarly, the microstructure morphology of Vantablack coatings is critical for achieving their exceptional light-absorbing properties [10,11].

Table 1 reports a comparison of black colours published in the recent literature. Commercial black pigments are derived from cobalt ferrochromite (DCMA 13-40-9) and nickel ferrochromite (DCMA 13-50-9), though it is easy to find industrial pigments using other cations, such as Mn. The usual L*a*b* parameters of ferrochromite commercial powder are around 41.0/1.0/1.0 that improve when dispersed in glaze (5 wt%) to values around 33.0/1.0/0.0 with an estimated reflectance in visible range R_Vis_ of 3.5 and 4.5% in powder and glaze, respectively [14,15].

In a recent study [11], the authors investigated the potential to develop intense black ceramic pigments comparable to, or surpassing, carbon black (L*a*b* = 20.2/0.1/0.1; visible, near-infrared, and total reflectance values R_Vis_/R_NIR_/R = 3/3/3%). Carbon black is widely regarded as the reference standard for black inorganic pigments used in paints; however, its poor thermal stability makes it unsuitable for high-temperature ceramic applications.

This goal could be achieved using various cuprates based on spinels, such as CuCr_2_O_4_ [16] or mcconnellite [17]. The tetragonal distorted spinel CuCr_2_O_4_ improves the black colour of ferrochromites, showing the powder L*a*b* = 40.5/0.1/−0.1 and R_Vis_/R_NIR_/R = 4.2/3.4/3.8, which improves when was 0.5 wt% glazed in soda-lime glass (800 °C) (L*a*b* = 26.3/−0.3/−0.5 and R_Vis_/R_NIR_/R = 3.8/4.5/4.1), reflecting the 96.2% of visible light incident. Likewise, exploring the effect of microwave synthesis on cuprates, using dielectric firing assisted by microwaves (60 min at 800 W), mcconnellite-synthesized pigment powder shows a good black shade (L*a*b* = 36.5/−4.8/−0.8 and R_Vis_/R_NIR_/R = 9.9/11.2/10.5), and 5 wt% glazed in a zinc-free ceramic glaze with maturation point at 1000 °C produces an intense coloured black glaze surface with L*a*b* = 11.3/−0.6/−3.0, associated with R_Vis_/R_NIR_/R = 1.4/1.9/1.6, thus reflecting 98.6% of the visible light, which borders on ultrablack behaviour. The microstructure of powder prepared by fast dielectric firing and the nature of the glaze enhances the black yield of the glassy coating. Likewise, the presence of zinc in the glaze composition should be avoided, because it reacts with the pigment to produce high stability zinc spinel such as gahnite ZnAl_2_O_4_ or ZnCr_2_O_4_ and exsolve copper (II) in the glassy matrix, creating a shift in colour to green-blue shades [16,17]. Other cuprates such as CuFe_5_O_8_ are codoped with 0.2 molar of MnO_2_ and 0.75 molar of Ni_2_O_3_; using 6.25% of MgO as a mineraliser produces black powders of L*a*b* = 21.36/1.10/−0.24 and 5 wt% when glazed at 1000 °C (with 5.12 wt% of ZnO in its composition). This improves the black colour to L*a*b* = 16.95/−0.18/−0.12, which is lower than that obtained by the simple mcconnellite but better than that of CuCr_2_O_4_ and ferrochromite spinels [18]. Four other important black pigments, such as Cr–hematite to Mn–melilite, were utilized for glazes in [19] and are also included in Table 1. The chromium-doped hematite with R_Vis_ of 4 and 4% in powder and glaze at 1000 °C, respectively, and the perovskite YMnO_3_ with R_Vis_ of 3 and 7% in powder and glaze at 1000 °C, respectively, stood out. Therefore, from the literature data, delafossite cuprates appear as possible candidates to produce ultrablack ceramic pigments.

In this study, the use of other delafossite cuprates, including CuMnO_2_ and CuFeO_2_, as well as iron- and manganese-doped mcconnellite composites, was investigated as potential candidates for producing intense black ceramic pigments. The materials were synthesized via conventional solid-state methods and fired using both electric kilns and fast dielectric (microwave) furnaces, with mcconnellite (CuCrO_2_) employed as the reference pigment. To the best of our knowledge, the synthesis, characterization, and application of cuprate delafossites (crednerite, delafossite, and mcconnellite) as ceramic pigments in glazes aimed at achieving ultrablack (R_Vis_ < 1%) or deep-black (R_Vis_ < 3%) surfaces have not previously been reported.

## 2. Materials and Methods

Delafossite CuMO_2_ (M = Mn,Fe,Cr) pigments were synthesized by solid-state method using tenorite CuO, eskolaite Cr_2_O_3_, bixbyite Mn_2_O_3_, and hematite Fe_2_O_3_ oxides as precursors, with a particle size of 0.3–5 µm (supplied by QUIMIALMEL SA, Castelló, Spain, 99.8 wt% purity). The precursors were mechanically homogenized in an electric grinder (20,000 rpm) for 5 min and then fired at 1100 °C for 3 h in electric kiln and also using fast microwave-assisted dielectric firing [20,21].

This hybrid microwave-assisted heat treatment of solid reactant mixtures is conducted in a closed kiln internally coated with a susceptor, which functions as an external heater during the initial stage of microwave irradiation [22]. Upon exposure to microwaves, the susceptor rapidly increases in temperature and transfers heat to the reactant mixture by radiation and/or conduction. Once the mixture reaches the critical temperature of the irradiated material, it begins to absorb microwave energy directly and continues heating autonomously.

In this study, dielectric firing was performed using a conventional microwave equipment operating at 900 W and 2.45 GHz, equipped with a cylindrical kiln for preheating the reactant mixture. The kiln (inner dimensions: height 4.5 cm, diameter 13.5 cm) was constructed from aluminosilicate fibreboard (density: 0.3 g cm^−3^), a material transparent to microwaves with penetration depths of 0.007 m at room temperature and 0.0025 m at 1300 °C. The kiln interior was coated with α-SiC (hexagonal, density: 3.2 g cm^−3^) serving as a susceptor (Glass Fusing & Cutting Tools, Tianjin, China; product code 1282151), as previously described in [17]. The temperature during firing was estimated using Process Temperature Control Rings, M.E. SCHUPP, Aachen, Germany (PTCR, ETH type, 850–1100 °C). Based on PTCR shrinkage measurements taken with a micrometre, the estimated temperatures reached approximately 950 °C after 20 min, 1050 °C after 30 min, and 1150 °C after 60 min of irradiation.

X-ray Diffraction (XRD) was performed on a Siemens D5000 diffractometer using Cu K_α_ radiation (10–70°2θ range, scan rate 0.03°2θ, 5 s per step and 40 kV and 20 mA conditions). Additional XRD processes with the addition of 30 wt% of α-Al_2_O_3_ as an internal measurement pattern (10–70°2θ range, scan rate 0.02°2θ, 10 s per step and 40 kV and 20 mA conditions) were performed for cell parameters estimations. The lattice constants were determined from corrected peak positions using α-Al_2_O_3_ as standard and least square refinement programmes.

The L*a*b* colour parameters of samples were measured according to the CIE-L*a*b* (Commission Internationale de l’Éclairage) [23], using an X-Rite SP60 spectrometer with standard lighting D65 and a 10° observer. L* measures the lightness (100 = white, 0 = black) and a* and b* the chromatic components (−a* = green, +a* = red, −b* = blue, +b* = yellow).

The tolerance ΔE* (based on the L*a*b* parameters), is evaluated using Equation (1):

(1) $$\Delta E*=\sqrt{{\Delta L*}^{2}+{\Delta a*}^{2}+{\Delta b*}^{2}}$$

UV–Vis–NIR spectra of the samples were recorded using a Jasco V-670 diffuse reflectance spectrophotometer. The optical band gaps were estimated using the Tauc method [24]. For the optimized pigments, optical reflectance spectra were collected over the 350–2500 nm range. The total solar reflectance (R), the solar reflectance in the near-infrared range (R_Nir_), and the solar reflectance in the visible range (R_Vis_) were evaluated from the UV–Vis–NIR diffuse reflectance spectra. These parameters were calculated as the ratio between the integral of the measured spectral irradiance multiplied by the solar irradiance and the integral of the solar irradiance over the same wavelength interval, namely 350–2500 nm for R, 700–2500 nm for R_NIR_, and 350–700 nm for R_Vis_, as expressed in Equation (2):

(2) $$R=\frac{\int_{350}^{2500}r\left(\lambda \right)i\left(\lambda \right)d\lambda }{\int_{350}^{2500}i\left(\lambda \right)d\lambda }$$

where *r*(*λ*) is the dimensionless spectral reflectance measured from UV–Vis–NIR spectroscopy and *iλ*) is the standard solar irradiance (Wm^−2^nm^−1^), according to the American Society for Testing and Materials (ASTM)’s Standard G173-03 [25] The solar absorption spectrum of the samples, representing their wavelength-dependent absorption relative to solar irradiance, was calculated by evaluating the numerator and denominator of the equation at each wavelength.

The pigmenting capacity of the samples was evaluated by incorporating the pigment into a double-firing frit, zinc-free, with a maturation point of 1000 °C (composition in wt%: SiO_2_ 72, Na_2_O 14, K_2_O 1.5, CaO 9, Al_2_O_3_ 2.3; average grain size: 3–20 μm; supplied by Torrecid S.A., l’Alcora, Spain). Glazed samples were prepared on white stoneware tile substrates. The glaze was manually mixed with the pigment and water in a weight ratio of 100:5:40 using an agate mortar and applied to a thickness of approximately 1500 μm using the Doctor Blade technique. A 40% shrinkage of the glaze layer was observed after firing.

Molten glazes interact with pigment particles through dissolution or chemical reactions, potentially leading to pigment degradation or colour alteration. The aggressiveness of the glaze depends on both its chemical composition and the firing temperature. The Dry Color Manufacturers Association classifies pigment powders into three categories [26,27]:

- *Category A* deals with pigments suspended in glass matrixes which require the highest degree of heat stability and chemical resistance to withstand the attack of molten glass.
- *Category B* deals with pigments suspended in plastics and other polymers, which require only moderate heat stability.
- *Category C* deals with pigments suspended in liquid vehicles, which require little, or no heat stability.

Ceramic pigments belong to Category A, showing stability and chemical resistance to molten ceramic glazes: Vantablack and Carbon Black belong to Category C and B, respectively.

In the field of polymers, Carbon Black is the reference standard for deep-black shades. The Industry Tint Reference Black (ITRB) is a standardized Carbon Black material used to evaluate the tinting strength of other Carbon Black pigments in polymers, following ASTM D3265 [28] or ISO 5435 [29] test methods. These methods involve mixing the black standard with zinc oxide and a liquid organic plasticizer. The resulting paste is mulled, homogenized, and spread as a layer of uniform thickness. Its light reflectance is then measured with a reflectometer and compared to a paste prepared with the sample under investigation.

Three specific metrics are used to quantify the degree of blackness based on tristimulus values ((X, Y, Z) for the sample and (Xn, Yn, Zn) for the reference): (a) blackness (My = 100 log_10_(Yn/Y)) measures the overall degree of blackness and is directly related to reflectance; (b) jetness or darkness (Mc = 100 [log_10_(Xn/X) + log_10_(Yn/Y) − log_10_(Zn/Z)]) reflects the colour-dependent black value, influenced by primary particle size, surface area, and dispersion in the polymer; and (c) undertone (dM = Mc − My) indicates whether the black exhibits a brown-reddish undertone (dM < 0), is neutral (dM = 0), or shows a bluish undertone (dM > 0), the latter often being preferred [30,31]. These metrics, however, are not directly applicable to ceramic pigments, which must remain stable at high temperatures when incorporated into ceramic glazes.

## 3. Results and Discussion

### 3.1. Characterization of Powders: Effect of Microwaves Firing

**Figure 1:**
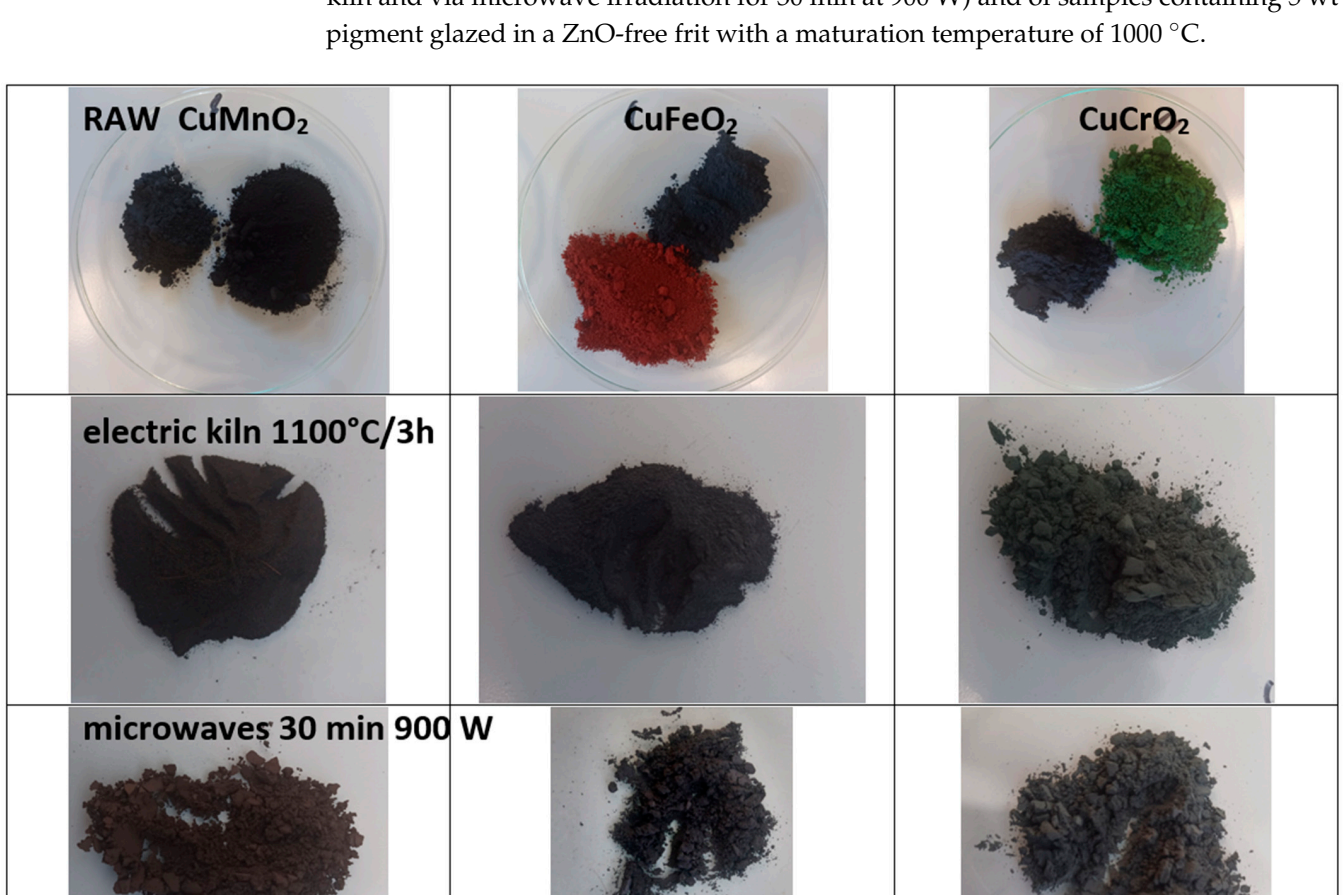
Powders before mixture and fired in electric kiln and with microwaves at 900 W for 30 min).

Figure 1 and Table 2 summarize the properties of the powders (fired in an electric kiln and via microwave irradiation for 30 min at 900 W) and of samples containing 5 wt% pigment glazed in a ZnO-free frit with a maturation temperature of 1000 °C.

All powders fired in the electric furnace exhibited a dark black appearance. However, subtle undertones were observed: manganese crednerite displayed a reddish-brown hue (a* > 0), iron delafossite powders exhibited a bluish undertone (b* < 0), and mcconnellite showed a greenish undertone (a* < 0). The visible-range reflectance (R_Vis_) follows the sequence 1MnCE < 3CrCE < 2FeCE, corresponding to a blackness order of 1MnCE > 3CrCE > 2FeCE. This trend is consistent with the increasing band gap (in the NIR range) and decreasing ΔE* values with increasing blackness (Table 2).

For microwave-fired powders, the R_Vis_ sequence differs (1MnMW < 3FeMW < 2CrMW) and is associated with increasing ΔE*, although the band gap values are comparable to those of the electrically fired samples and follow the same sequence. Microwave firing reduced R_Vis_ for the Mn and Fe samples, enhancing their blackness, whereas the Cr sample exhibited a slightly higher R_Vis_ under microwave firing compared to the electric-fired sample.

**Figure 2:**
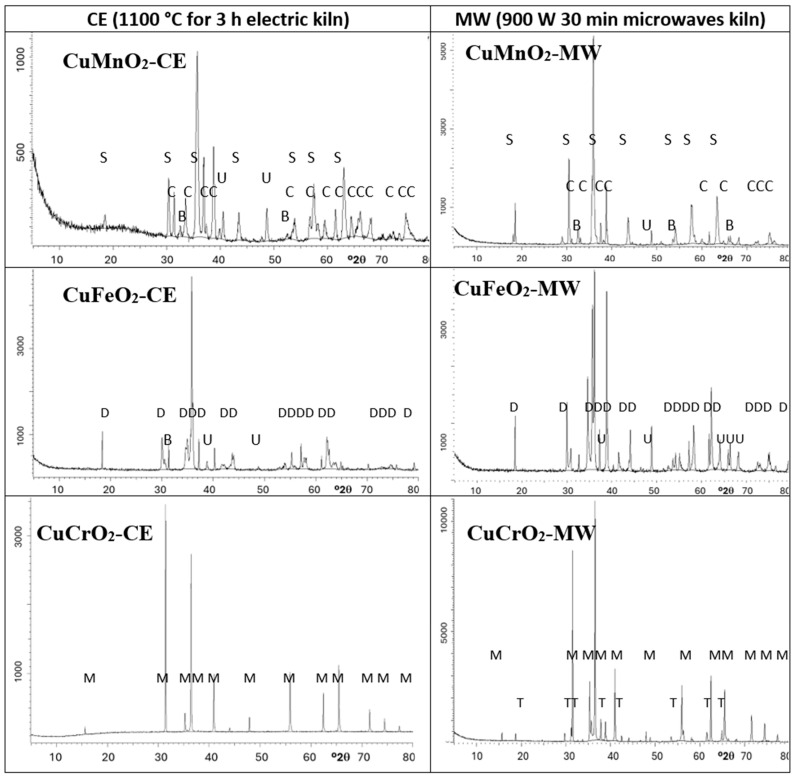
XRD of powders. Crystalline phases: S (CuMn_2_O_4_), C (CuMnO_2_ crednerite), D (CuFeO_2_ delafossite), M (CuCrO_2_ mcconnellite), U (CuO), B (Mn_2_O_3_), and T (CuCr_2_O_4_). (the abscissa of each diffractogram is °2θ (2 degrees theta) and the ordinate is “counts”).

The XRD diffractograms of samples are shown in Figure 2 and the crystalline phases identified are summarized in Table 3.

In the case of crednerite (CuMnO_2_), the spinel phase CuMn_2_O_4_ crystallizes alongside monoclinic crednerite under conventional electric kiln firing. However, when synthesized using a microwave kiln (1MnMW), crednerite exhibits very weak XRD peak intensities. Residual peaks corresponding to CuO and Mn_2_O_3_ are also detected in both samples. The observed phase composition aligns with the Cu–Mn–O phase equilibria [32], where crednerite is metastable at room temperature and its formation is strongly dependent on the heat treatment of the oxide mixture. As reported in [33,34,35], the XRD pattern of CuMnO_2_, prepared via low-temperature exchange reactions and essentially free of impurities, can be indexed to a monoclinic crednerite structure (space group *C2/m*). Thermogravimetric analysis (TGA) reveals an oxygen uptake, resulting in a reversible transition to the oxygen-rich spinel Cu_x_Mn_2–x_O₄ (x ≈ 1.03). Subsequent firing above 940 °C induces oxygen loss, converting the oxide back to Cu_1.1_Mn_0.9_O_2_, with residual spinel remaining as an impurity. At 1150 °C, CuMnO_2_ undergoes peritectic melting, and XRD indicates the coexistence of cubic spinel and CuO/Cu_2_O phases. Consistently, sample 1MnCE was observed to melt after firing at 1200 °C for three hours.

The indexing of monoclinic crednerite CuMnO_2_ (*C2/m* ICDD 50-0860) diffraction peaks for 1MnCE indicates a unit-cell volume slightly larger than previously reported values (V = 93.5(3) Å^3^, compared with 92.5 Å^3^ by Trari et al. [33] and 92.1 Å^3^ in the literature [34,35]) (Table 4). CuMnO_2_ forms a solid solution of the general composition Cu^+^_1−x−2y_Cu^2+^_y_Mn^3+^_1−x_Mn^4+^_x_O_2_, with cationic defects located within O–Cu–O dumbbells. In copper manganite, CO conversion is governed by the redox equilibrium Cu^+^ + Mn^3+^ ⟷ Cu^2+^ + Mn^4+^, where CO chemisorbs on Cu^2+^/Mn^4+^ sites and O_2_ on Cu^+^/Mn^3+^ sites [36]. The ionic radius of Cu^+^ (0.60 Å, CN = 2) in O–Cu–O dumbbells is larger than that of Cu^2+^, whereas Mn^3+^ in octahedral coordination (0.72 Å for low spin; 0.785 Å for high spin) is larger than Mn^4+^ (0.67 Å) [37]. Consequently, the substitution of Cu^2+^ into O–Cu–O sites, accompanied by the reduction of Mn^4+^ to Mn^3+^ in octahedra, leads to lattice expansion, producing a net dilation consistent with the experimentally observed unit-cell volume. The indexing of peaks of spinel CuMn_2_O_4_ as cubic phase (*Fd3m* ICDD 74–2422) for 1MnCE and 1MnMW gives a cell parameter of a = 8.308(1) Å, which is very close to the literature data [38] (Table 4).

The different compositions of the samples, with a composite of spinel and crednerite undergoing the electric firing and the spinel as the only phase undergoing microwave firing, explains the slight decrease in R_Vis_ in the microwave-fired sample (5.47 and 4.85% for 1MnCE and 1MnMW, respectively), indicating the lower black colour of the crednerite CuMnO_2_ than that of the CuCr_2_O_4_ spinel. However, additional factors may also be involved, such as the so-called “composite effect” arising from pigments composed of different elements and compounds. This effect can modify the reflection, absorption, and scattering of light, thereby generating distinct colour characteristics. Furthermore, the size and morphology of pigment particles influence their optical behaviour: larger particles generally enhance light scattering, whereas smaller particles promote greater light absorption. In this case, the composite effect may contribute to the enhanced blackness of 1MnCE, while the smaller particle size of 1MnMW) likely intensifies its black appearance. The combined influence of these factors accounts for the slightly increased blackness observed in 1MnMW.

For delafossite CuFeO_2_, both samples display intense peaks corresponding to the delafossite phase (space group *R-3m*). In contrast to the electrically fired sample (2FeCE), which exhibits only very weak peaks of unreacted oxides, the microwave-fired sample (2FeMW) shows pronounced peaks corresponding to tenorite (CuO). The indexing of the delafossite peaks (ICDD 75-2146; Table 4) reveals lattice parameters slightly lower than those reported in the literature [39]. Specifically, the cell volume of the delafossite in 2FeCE is marginally smaller than that of 2FeMW (V = 136.0(3) and 135.8(3) Å^3^ for 2FeMW and 2FeCE, respectively, compared to 136.8668 Å^3^ in reference [39]).

The composite effect in sample 2FeMW (CuO intimately mixed with CuFeO_2_), along with the possibly lower blackness of delafossite compared with tenorite CuO, can explain the decrease in R_Vis_ for the microwave-fired powder (6.87% and 6.45% for 2FeCE and 2FeMW, respectively).

For the reference mcconnellite, XRD analysis shows that CuCrO_2_ crystallizes as the predominant phase in both electrically fired (3CrCE) and microwave-fired (3CrMW) samples. In the electrically fired 3CrCE, only mcconnellite peaks are observed. In contrast, the microwave-fired 3CrMW exhibits weak peaks correspond to a tetragonally distorted spinel, CuCr_2_O_4_. The indexing of the mcconnellite peaks (space group *R3m*, ICDD 39-0247; Table 4) indicates lattice parameters slightly higher than those reported in the literature [40,41]. Specifically, the cell volume of mcconnellite in 3CrMW is marginally larger than in 3CrCE (V = 127.9731 Å^3^ in reference [40,41], 129.7(4) Å^3^ for 3CrMW, and 129.9(5) Å^3^ for 3CrCE).

The high content of black-coloured mcconnellite in the 3CrCE sample explains the slight increase in R_Vis_ (i.e., a decrease in blackness) in the microwave-fired sample (R_Vis_ = 6.10% and 6.98% for 3CrCE and 3CrMW, respectively)

**Figure 3:**
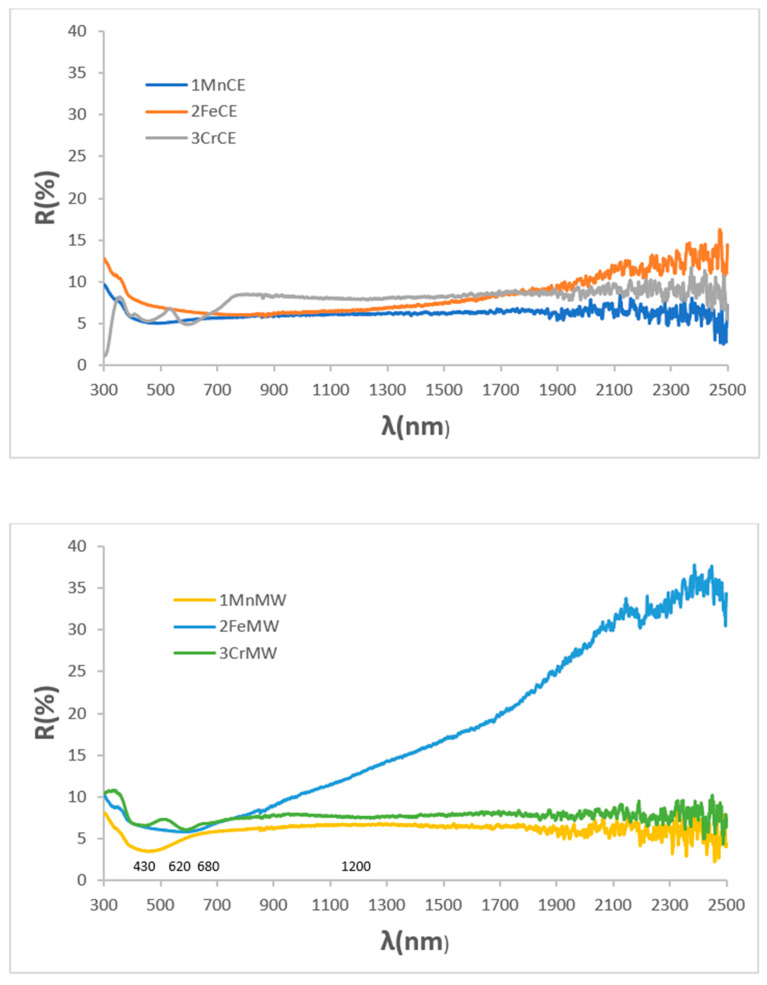
UV–Vis–NIR reflectance spectra of powders.

Figure 3 presents the UV–Vis–NIR diffuse reflectance spectra of the powders. Both manganese-containing samples exhibit nearly featureless spectra, with only a very weak band at 310 nm and an additional reflectance minimum at 430 nm for the microwave-fired 1MnMW sample. Similarly, the iron delafossite powder (2FeCE), which consists predominantly of CuFeO_2_, shows a mostly featureless spectrum with a weak minimum at 310 nm. In contrast, the microwave-fired 2FeMW powder displays a pronounced increase in reflectance in the NIR region, consistent with the presence of CuO detected by XRD. The reference mcconnellite exhibits largely featureless spectra in both firing conditions, with weak minima (corresponding to absorbance maxima), observed at 430, 620, 680, and 1200 nm. These features are attributed to Cr^3+^ (d^3^) electronic transitions in octahedral coordination: ^4^A_2_g(^4^F) → ^4^T_1_g(^4^F) at 430 nm, ^4^A_2_g(^4^F) → ^4^T_2_g(^4^F) at 680 nm, and ^4^A_2_g(^4^F) → ^2^E_g(^2^G) at 1200 nm. The absorption at 620 nm is assigned to low-coordinated Cu^+^ ions in the O–Cu^+^–O dumbbells of the mcconnellite structure [42].

**Figure 4:**
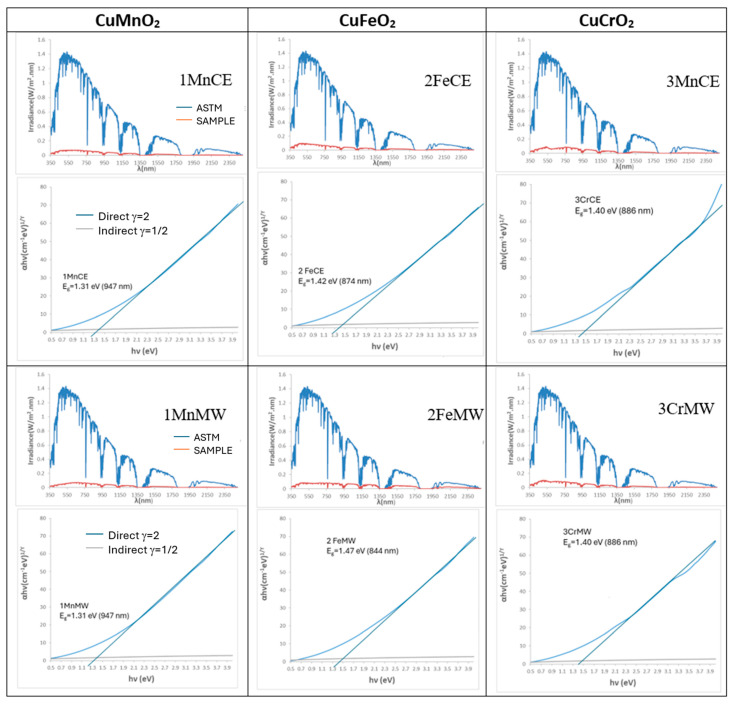
Tauc’s plot and solar spectra of powders.

Figure 4 presents the solar reflectance spectra and Tauc plots of the powders. The band gaps are identical for the crednerite and mcconnellite samples under both electric- and microwave-firing conditions (1.31 eV and 1.40 eV, respectively). In contrast, the iron delafossite samples exhibit different band gaps due to the presence of CuO in the microwave-fired 2FeMW sample. The solar spectra highlight the strong absorption of solar radiation by all intense black powders across both the visible and near-infrared regions.

**Figure 5:**
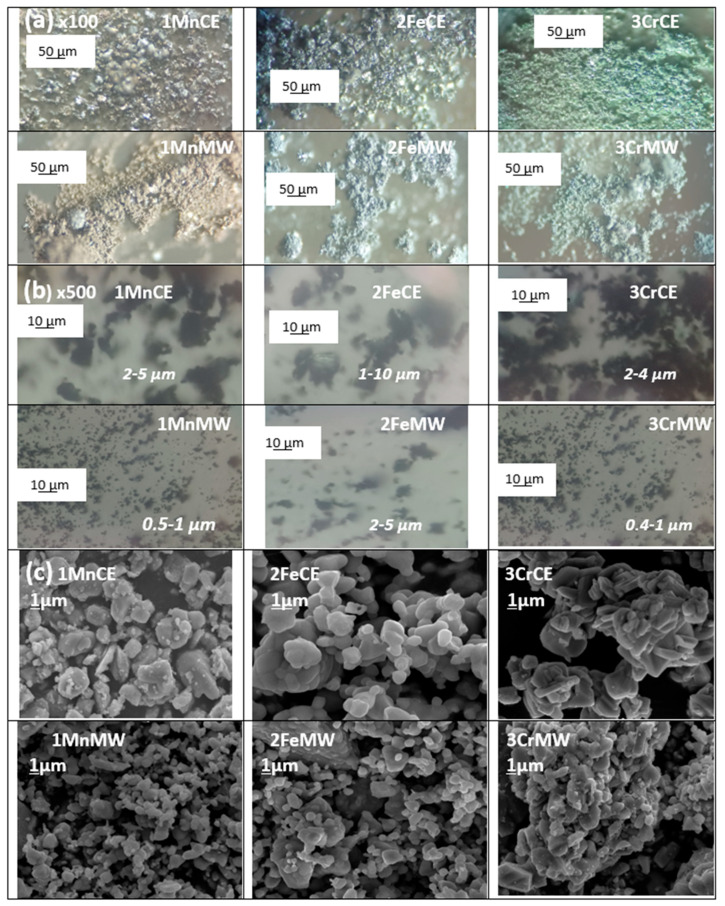
(**a**) Optical images of powders ×100; (**b**) Optical images of powders ×500; (**c**) SEM micrographs of powders (the segment below the value in microns is the corresponding length).

Figure 5 shows optical images of the powders at two magnifications (×100 and ×500) alongside SEM micrographs. The ×100 optical images reveal that manganese crednerite exhibits a brown undertone, iron delafossite a bluish undertone, and mcconnellite a greenish undertone, consistent with the L*a*b* colour parameters. The analysis of the ×500 optical images and SEM micrographs allows the estimation of particle size, indicating that microwave firing produces smaller particles in all cases compared to electric firing. Particle size has a moderate influence on the blackness of the powders: R_Vis_ decreases slightly for manganese and iron powders and for mcconnellite in the microwave-fired samples. The effect of particle size is expected to be more pronounced in glazed samples, as smaller particles increase the number of colour points per unit volume within the glassy matrix, enhancing the perceived blackness.

### 3.2. Characterization of 5 wt% Glazed Samples in Double Firing Frit with Maturation Point at 1000 °C, Zinc-Free, Using Electric Firing for Glazing

**Figure 6:**
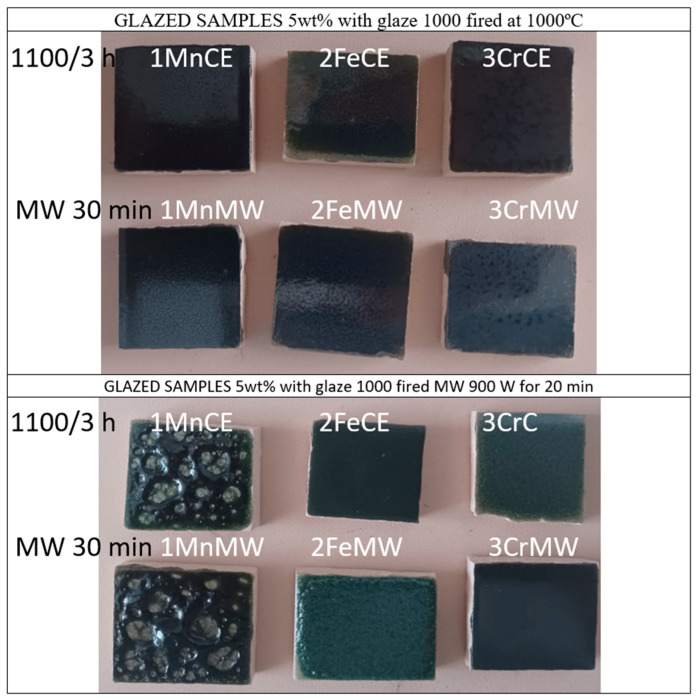
Images of 5 wt% glazed samples in ZnO-free frit of 1000 °C.

Figure 6 and Table 2 show the characteristics of 5 wt% glazed powders in double firing frit with maturation point at 1000 °C, zinc-free, using an electric kiln for glazing.

All electrically fired glazes appear dark black. Manganese- and iron-containing glazes exhibit a bluish undertone (b* < 0), whereas mcconnellite shows a neutral to greenish hue (a* ≤ 0). Among glazes derived from electrically fired powders, R_Vis_ decreases in the sequence 1MnCE < 2CrCE < 3FeCE, reflecting the blackness trend 3CrCE > 2FeCE > 1MnCE and the corresponding decline in ΔE* (Table 1). Mcconnellite stands out with an intense black tone (R_Vis_ = 4.40, ΔE* = 4.6) and a higher measured bandgap than the original powder.

For glazes derived from microwave-fired powders, the R_Vis_ sequence follows 2FeMW > 1MnMW > 2CrMW, consistent with the decreasing trends of both ΔE* and bandgap. Mcconnellite is notable for its deep-black appearance (R_Vis_ = 1.41, ΔE* = 3.5), accompanied by a reduced bandgap relative to the corresponding powder. Overall, Mn- and Cr-containing glazes from microwave firing exhibit lower R_Vis_ values than their electrically fired counterparts, indicating a deeper black shade. In contrast, the Fe sample shows a higher R_Vis_ under microwave firing, which is attributed to the presence of CuO in the powder.

**Figure 7:**
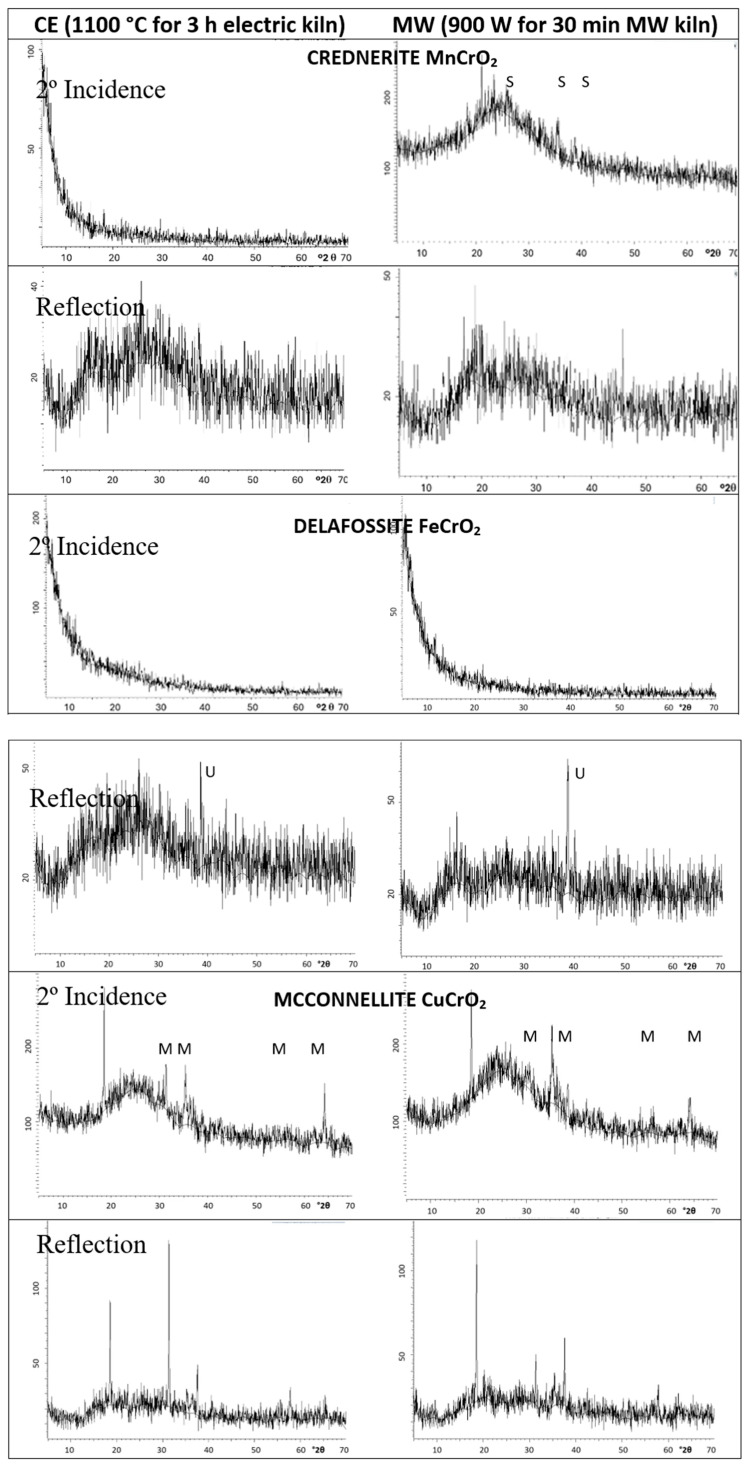
XRD low-incidence angle (2°) and conventional backscattering of 5 wt% glazed samples in frit fired with electric kiln. Crystalline phases: S (CuMn_2_O_4_), M (CuCrO_2_ McConnellite), U (CuO tenorite). (the abscissa of each diffractogram is °2θ (2 degrees theta) and the ordinate is “counts”).

Figure 7 presents the XRD diffractograms of the samples, recorded under both backscattering and grazing-incidence conditions (2° incidence angle). X-ray diffraction (XRD) analyses were carried out using a Siemens D5000 diffractometer with Cu Kα radiation over the 2θ range of 10–70°, employing a step size of 0.02° 2θ and a counting time of 10 s per step, under operating conditions of 40 kV and 20 mA. These parameters enable the assessment of pigment particle behaviour dispersed within the amorphous silica glassy matrix, which contributes to the black coloration of the samples.

The X-ray diffraction intensity of crystalline phases detected in glazed samples depends on several factors, including the atomic number (elements with higher atomic numbers possess greater scattering power; in this case, Cr [Z = 24], Mn [Z = 25], and Fe [Z = 26] exhibit comparable scattering ability), the degree of crystallinity (higher crystallinity increases the number of atoms contributing to diffraction), crystal size (larger crystals yield stronger diffraction peaks), and the crystallographic orientation of the crystal relative to the incident X-ray beam [43]. Therefore, the detection of crystalline phases within glazes sharing a similar glass matrix, comparable atomic numbers, and equivalent beam orientation requires that particles exceed certain thresholds of concentration and size. Finely dispersed and/or low-concentration crystalline phases, particularly those affected by glass corrosion, may fall below the detection limit of the XRD technique.

For crednerite (CuMnO_2_), the grazing-incidence XRD of the glazed microwave-fired powder 1MnMW reveals peaks corresponding to the spinel phase CuMn_2_O_4_. In contrast, no peaks or crystallization halos are detected in the glazed 1MnCE powder. Backscattering XRD shows a double crystallization halo between 20° and 25° 2θ, attributed to the silica glass matrix. Crednerite is not observed after interaction with the molten glaze; thus, the highly stable spinel CuMn_2_O_4_ is identified as the crystalline phase responsible for the black coloration.

For delafossite (CuFeO_2_), grazing-incidence XRD shows no detectable response. Conventional backscattering XRD displays crystallization halos at ~20° and 25° 2θ, along with a diffraction peak at 39° 2θ, corresponding to the intense [114] reflection plane of tenorite (CuO). This indicates that delafossite decomposes during glazing, and the black coloration cannot be attributed to CuFeO_2_, but rather to the concentration of CuO. It is well established that Cu^2+^ ions dissolved in glazes derived from copper-based pigments can produce either a green or a blue coloration, depending on their coordination environment: octahedrally coordinated Cu^2+^ in the glassy matrix is associated with green hues, while square-planar coordinated Cu^2+^ surrounded by oxygen atoms in the glass network yields blue tones [44,45]. In the present case, however, the behaviour differs. The 1FeCE powder crystallizes predominantly as delafossite, with weak peaks of CuO phase, whereas the 1FeMW powder exhibits pronounced CuO diffraction peaks. According to the literature, delafossite is thermally unstable and decomposes into CuO at approximately 1070 °C [46]. These results therefore indicate that microwave irradiation accelerates the decomposition of delafossite into CuO. In the glazed samples, however, the low-incidence XRD patterns are nearly identical for both cases, showing the intense [114] reflection plane characteristic of tenorite (CuO) in each. Both glazed samples also exhibit a greenish hue, consistent with the partial dissolution of copper into the glaze. These results indicate that delafossite is unstable and decomposes into CuO within the glassy matrix, following a mechanism analogous to its thermal decomposition at elevated temperatures.

For the reference mcconnellite, XRD confirms the crystallization of CuCrO_2_ in both glazes prepared from electric- and microwave-fired powders, analyzed by conventional and grazing-incidence XRD. The resistance of mcconnellite to molten glass highlights the high stability and effectiveness of CuCrO_2_ as a black ceramic pigment.

**Figure 8:**
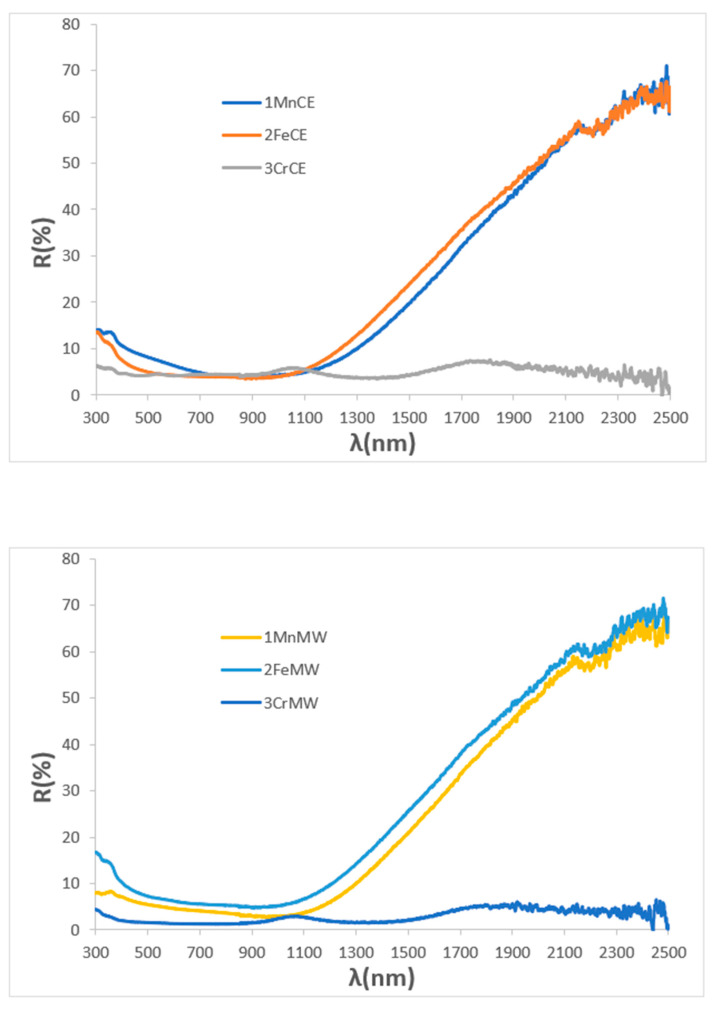
UV–Vis–NIR reflectance spectra of samples glazed using electric kiln.

Figure 8 shows the UV–Vis–NIR reflectance spectra of samples glazed in an electric kiln. Consistent with the XRD results, only mcconnellite, which remains stable against attack by the molten glaze, exhibits a flat reflectance across the Vis–NIR range. In contrast, manganese- and iron-containing delafossites, which are unstable in the glaze medium and crystallize as spinel CuMn_2_O_4_ and CuO, respectively, show increased reflectance in the NIR region, a feature characteristic of potential solar-selective absorber surfaces [17].

**Figure 9:**
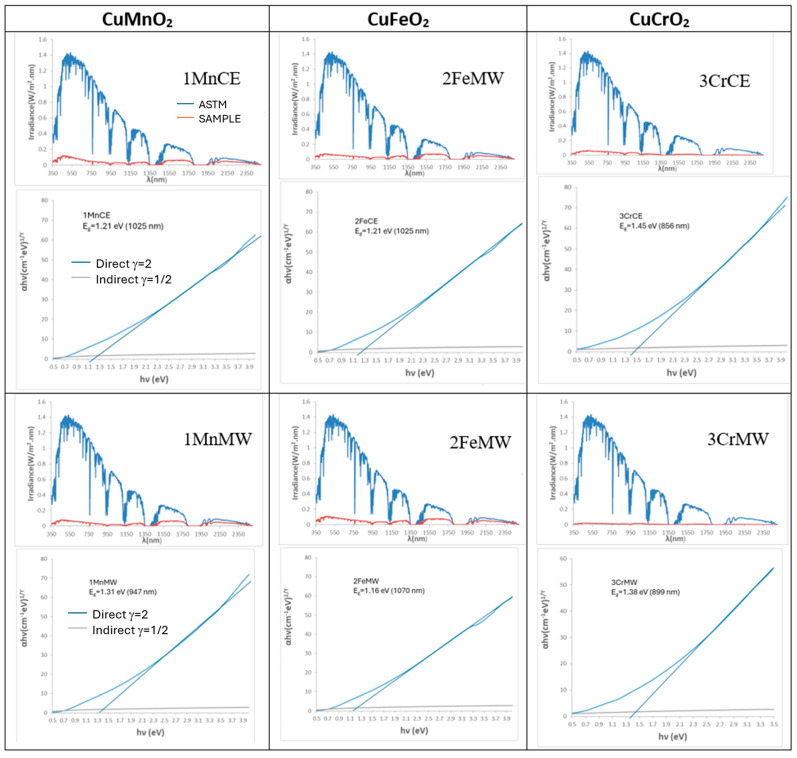
Tauc plots and solar spectra of glazed samples using electric kiln.

Figure 9 shows the solar spectra and Tauc plots of glazed samples prepared by electric firing. Bandgap values vary between samples from electrically and microwave-fired powders, reflecting the influence of different crystallization systems within the glass matrix. The spectra reveal strong solar absorption across the visible and near-infrared ranges, with sample 3CrMW exhibiting particularly high absorption, consistent with its deep-black colour (R_Vis_ = 1.41%), highlighting the effect of crystallization on optical performance.

### 3.3. Characterization of 5 wt% Glazed Samples in Double Firing Frit with Maturation Point at 1000 °C, Zinc-Free, Using Microwaves Firing for Glazing

Figure 6 and Table 2 summarize the characteristics of 5 wt% glazed powders in a double firing frit with a maturation point of 1000 °C, prepared using a microwave kiln.

All microwave-fired glazes exhibit a dark black appearance with pinhole defects, with the most pronounced in the manganese sample and also present in 2FeMW and 3CrCE, alongside a greenish undertone (a* ≤ 0). These features are attributed to the behaviour of Cu^2+^ ions within the glass: octahedrally coordinated Cu^2+^ produces a green hue, while square-planar coordination can yield blue coloration [44]. In the manganese and iron samples, the presence of Cu^2+^ compounds (CuMn_2_O_4_ and CuO) and the aggressive interaction with the molten glass promote the partial exsolution and reduction of Cu^2+^ to Cu^+^, releasing oxygen and resulting in the formation of the observed pinholes Equation (3) [44,45]:

2CuO_(glaze/green shade)_ → Cu_2_O_(glaze, red shade)_+ ½ O_2_ (3)

Crystalline phase stability dictates Cu^2+^ behaviour and optical properties: stable phases, as in mcconnellite, limit Cu^2+^ reduction and maintain blackness, whereas decomposed phases, such as crednerite and delafossite, promote Cu^2+^ reduction, resulting in greenish undertones and pinholes.

In microwave-glazed samples from electrically fired powders, R_Vis_ decreases in the order 1MnCE < 2CrCE < 3FeCE, corresponding to a blackness trend of 1MnCE > 2CrCE > 3FeCE, with higher ΔE* values linked to the greenish hue (b*) (Table 1).

**Figure 10:**
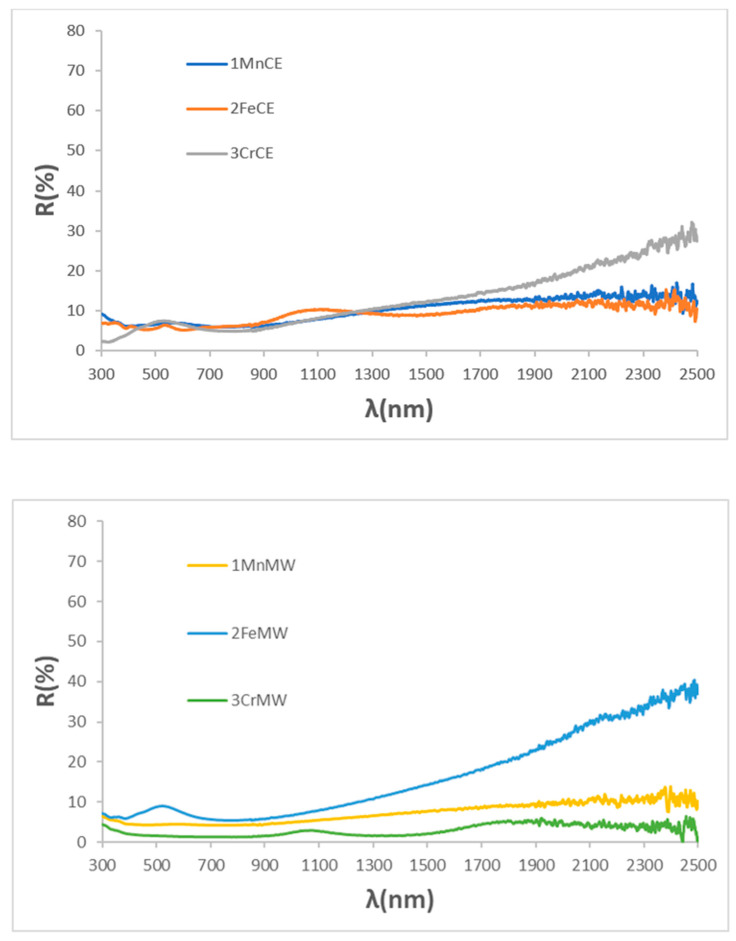
UV–Vis–NIR reflectance spectra of glazed samples using microwaves kiln.

For microwave-glazed samples, R_Vis_ follows the sequence 2FeMW > 1MnMW > 2CrMW, consistent with decreasing ΔE* and bandgap values. Mcconnellite exhibits an intense black appearance (R_Vis_ = 2.63, ΔE* = 4.3) and a reduced bandgap relative to the corresponding powder. Compared to electrically fired counterparts, microwave-glazed Mn and Cr samples show lower R_Vis_, indicating a deeper black shade, whereas Fe displays higher R_Vis_. UV–Vis–NIR spectra (Figure 10) reveal generally flat responses for microwave-glazed powders, with the Fe sample showing notable NIR reflectance. These findings suggest that microwave glazing promotes enhanced Cu exsolution from pigment particles while preserving their chemical stability better than electric kiln glazing.

**Figure 11:**
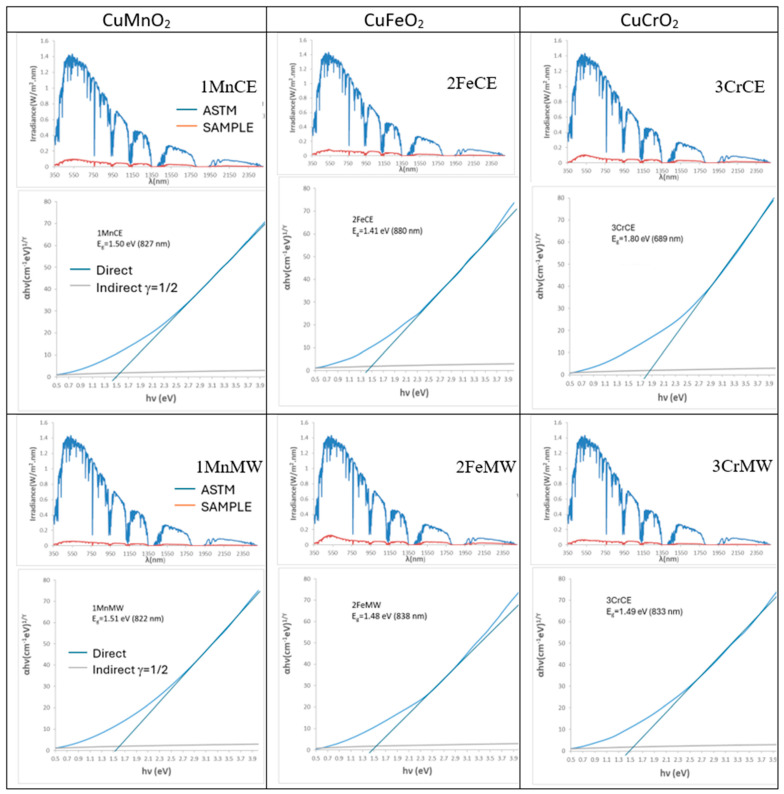
Tauc plots and solar spectra of glazed samples using microwaves kiln.

Figure 11 shows the solar spectra and Tauc plots of microwave-glazed samples. Bandgap values vary between powders fired electrically and by microwave, reflecting differences in crystallization within the glass matrix. The spectra indicate strong solar absorption across the visible and near-infrared ranges, with sample 3CrMW exhibiting particularly high absorption, consistent with its deep-black colour (R_Vis_ = 2.63), slightly higher than its electric kiln counterpart (R_Vis_ = 1.41).

### 3.4. Effect of Temperature and Microwaves Power in Blackness of Mcconnellite

Table 5 summarizes the properties of mcconnellite (CuCrO_2_) powders fired in an electric kiln at 1000 and 1100 °C for three hours and in a microwave kiln at 800 and 900 W for 30 min.

Mcconnellite is the only phase detected at both 1000 and 1100 °C, as shown in Figure 2 and reference [17]. However, the blackness of the powders increases (R_Vis_ decreases from 13.8 to 6.10), in agreement with the decrease in the green shade (b*), the bandgap, and the colour deviation ΔE* relative to Carbon Black.

Mcconnellite is the only crystalline phase detected at a microwave power of 800 W [17]. When the power is increased from 800 W to 900 W, the blackness of the powders increases (R_Vis_ decreases from 9.9 to 6.98). The weak diffraction peaks of spinel CuCr_2_O_4_ observed in this sample (Figure 2) indicate the presence of a “composite effect,” which alters the reflection, absorption, and scattering of light, thereby producing distinct colour responses and enhanced blackness.

Glazing in a 1000 °C double firing frit increases blackness for electric furnace powders (R_Vis_ 5.30 to 4.40), whereas microwave-fired powders show nearly constant or slightly higher R_Vis_ (1.40 to 1.41), likely reflecting the persistence of the spinel phase and the loss of composite scattering effects when particles are embedded in the glassy matrix.

### 3.5. Characterization of Mcconellite Doped with Iron and Manganese

To assess firing temperature, glaze compatibility, and the effects of Fe and Mn doping, three samples were prepared: mcconnellite (CuCrO_2_, reference), Fe-doped (Cu_0.8_Fe_0.2_)CrO_2_, and Fe–Mn co-doped (Cu_0.6_Mn_0.2_Fe_0.2_)CrO_2_. All were fired in an electric kiln at 1200 °C for three hours.

**Figure 12:**
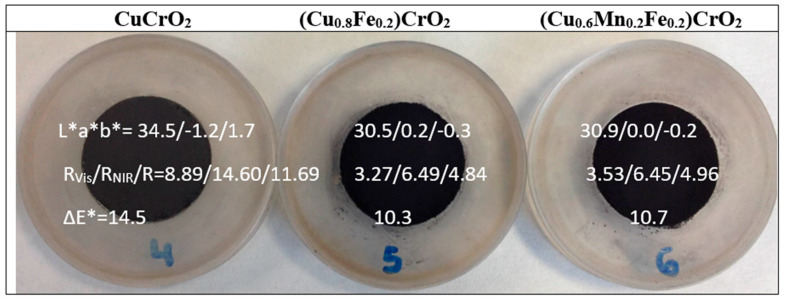
Powders fired at 1200 °C for 3 h of CuCrO_2_, Fe-doped (Cu_0.8_Fe_0.2_)CrO_2_, and Fe- and Mn-doped (Cu_0.6_Mn_0.2_Fe_0.2_)CrO_2_.

As shown in Figure 12, the fired powders appear black. Mcconnellite exhibits a slight greenish undertone (b* = −1.2), while the doped samples are nearly neutral. The undoped sample shows higher visible reflectance (R_Vis_ = 8.89%) than the doped powders (R_Vis_ ≈ 3%), corresponding to a deeper black colour.

**Figure 13:**
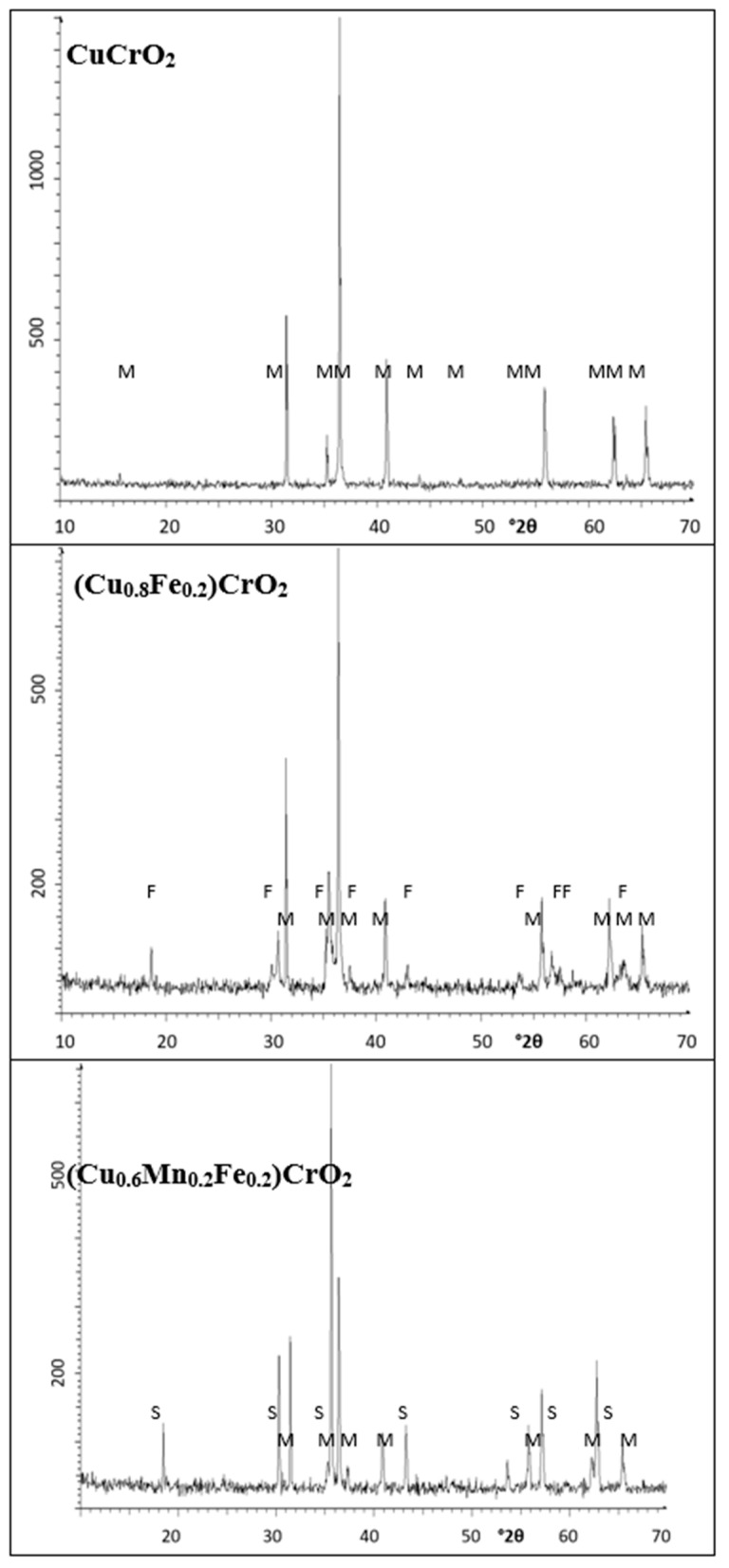
XRD of powders CuCrO_2_, Fe-doped (Cu_0.8_Fe_0.2_)CrO_2_, and Fe- and Mn-doped (Cu_0.6_Mn_0.2_Fe_0.2_)CrO_2_. Crystalline phases: M (mcconnellite CuCrO_2_), F (spinel Cu(Cr,Fe)_2_O_4_), S (spinel Cu(Cr,Fe,Mn)_2_O_4_).

Figure 13 shows the XRD patterns of the synthesized powders. The mcconnellite sample contains only the CuCrO_2_ phase. In the Fe-doped sample (Cu_0.8_Fe_0.2_)CrO_2_, mcconnellite remains predominant, with additional medium-intensity peaks corresponding to spinel Cu(Cr,Fe)_2_O_4_. In the Fe–Mn co-doped sample, the spinel Cu(Cr,Fe,Mn)_2_O_4_ phase dominates, accompanied by residual mcconnellite reflections. Spinels, known black pigments [14,15], contribute to increased light backscattering (composite effect), resulting in enhanced colour strength. Accordingly, the doped powders exhibit lower R_Vis_ values than pure mcconnellite.

**Figure 14:**
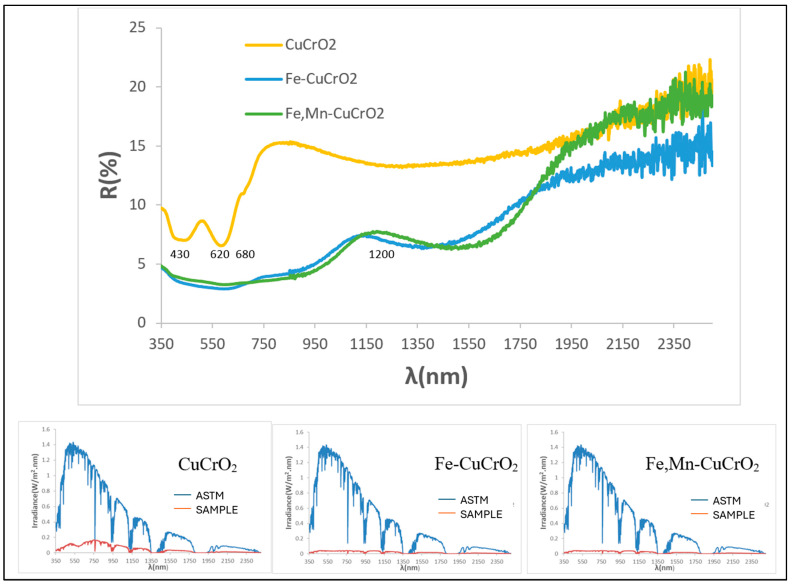
UV–Vis–NIR diffuse reflectance spectra of powders CuCrO_2_, Fe-doped (Cu_0.8_Fe_0.2_)CrO_2_ and Fe- and Mn-doped (Cu_0.6_Mn_0.2_Fe_0.2_)CrO_2_ and its respective solar spectra absorption.

Figure 14 presents the UV–Vis–NIR diffuse reflectance and the corresponding solar absorption spectra of powders. The mcconnellite shows higher reflectivity, consistent with its lower blackness. Characteristic absorption minima at 430, 620, and 1200 nm (Cr^3+^, octahedral coordination) and at 680 nm (Cu^+^) are clearly visible. In the doped samples, overlapping bands from Mn^3+^ and Fe^3+^ hinder individual band assignment. The solar absorption spectra confirm higher overall absorbance for the doped compositions across the solar range.

**Figure 15:**
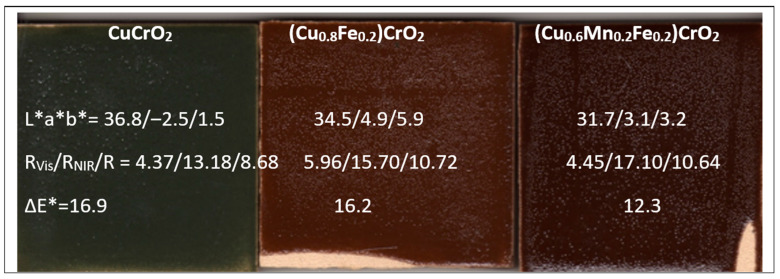
Images of 5 wt% glazed samples in glaze for porcelain (1190 °C) and alkyd paint (weight ratio paint/pigment/water = 7:2:5) of CuCrO_2_, Fe-doped (Cu_0.8_Fe_0.2_)CrO_2_, and Fe- and Mn-doped (Cu_0.6_Mn_0.2_Fe_0.2_)CrO_2_ and its respective solar spectra absorption.

Figure 15 shows images of 5 wt% glazed samples in a porcelain glaze (maturation point 1190 °C; composition in wt%: SiO_2_ 67, K_2_O 3, CaO 12.5, MgO 1.5, ZnO 6, Al_2_O_3_ 10; average grain size of 7–30 μm, supplied by Torrecid S.A. l’Alcora, Spain), including their colour and reflectance characteristics. The mcconnellite sample, having lost the composite effect observed in the powder form due to particle dispersion within the glassy matrix, now exhibits the deepest black shade with a slight greenish undertone (R_Vis_ = 4.37, b* = −2.5). In contrast, the doped samples display a visibly brown hue, with a* values of 4.9 and 3.1 for the Fe-doped and Fe–Mn co-doped compositions, respectively. When the pigments are glazed, the attack of this aggressive glassy matrix can dissolve and decompose the pigment particles, leading to the crystallization of highly stable spinels such as zinc spinels (ZnO should therefore be avoided in glazes to preserve pigment integrity) [44,45]. The high-temperature porcelain glaze supplied by Torrecid S.A. contains approximately 6 wt% ZnO; consequently, multicomponent brown spinels with a general composition of (Zn,Fe,Mn,Cu)(Fe,Mn,Cr)_2_O_4_ may form (see brown spinels in the CPMA classification [26]). These phases are difficult to analyze in glassy matrices containing dispersed particles.

**Figure 16:**
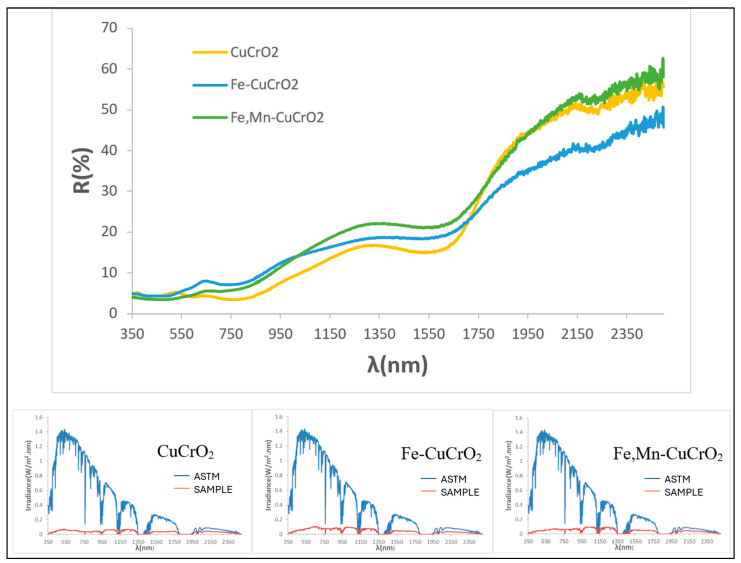
UV–Vis–NIR diffuse reflectance spectra of 5 wt% glazed samples in glaze for porcelain (1190 °C) CuCrO_2_, Fe-doped (Cu_0.8_Fe_0.2_)CrO_2_, and Fe- and Mn-doped (Cu_0.6_Mn_0.2_Fe_0.2_)CrO_2_ and its respective solar spectra absorption.

Figure 16 shows the UV–Vis–NIR diffuse reflectance spectra of 5 wt% pigmented glazes on porcelain (fired at 1190 °C) and their corresponding solar absorption spectra. The three samples exhibit similar spectral profiles; however, the mcconnellite-glazed sample shows slightly lower reflectance in the yellow–red wavelength region (550–700 nm), resulting in its visually black appearance, in contrast to the brown hues of the doped glazes. In all cases, reflectance in the NIR region increases, indicating that these glazed surfaces are promising candidates for selective solar absorber applications. Among them, mcconnellite demonstrates the highest absorption in the visible range [47].

## 4. Conclusions

Cuprate delafossite phases, such as CuMnO_2_ (crednerite) or CuFeO_2_, as well as iron- and manganese-doped mcconnellite composites, were analyzed as candidates for producing intense black ceramic pigments using conventional solid-state synthesis, employing both electric kiln and fast dielectric (microwave) firing, and compared with mcconnellite (CuCrO_2_) used as a reference.

In the case of crednerite, the electrically fired powder exhibits a composite of spinel and crednerite, whereas the microwave-fired powder consists almost exclusively of spinel, resulting in slightly lower blackness. When glazed in an electric kiln, spinel CuMn_2_O_4_ is detected in the microwave-glazed sample, while no diffraction peaks or crystallization halos are observed for the electrically fired sample. No evidence of crednerite preservation after interaction with the molten glaze is found. Therefore, the highly stable spinel CuMn_2_O_4_ is identified as the crystalline phase responsible for the black coloration in this system. Glazed samples prepared in a microwave kiln show identical behaviour.

For delafossite, both the electrically and microwave-fired powders contain a delafossite–tenorite (CuFeO_2_–CuO) composite, with weak tenorite peaks in the electrically fired sample. The so-called “composite effect” accounts for the slightly higher blackness of the tenorite-rich microwave-fired powder (R_Vis_ = 6.45% versus 6.87% for the electrically fired sample). After glazing in an electric kiln, conventional XRD patterns display broad crystallization halos together with a diffraction peak at 39° 2θ, corresponding to the intense [114] plane reflection of tenorite, indicating the decomposition of delafossite within the molten glass. Similar behaviour is observed for the samples glazed using a microwave kiln.

For mcconnellite, a single-phase material is obtained in the electrically fired powder, whereas the microwave-fired sample shows residual oxide peaks. The higher blackness of mcconnellite explains the slightly lower R_Vis_ value for the electrically fired sample (R_Vis_ = 6.10% versus 6.98% for the microwave-fired sample). Upon glazing in an electric kiln, both grazing-incidence and conventional XRD analyses confirm the crystallization of CuCrO_2_ in all cases. The persistence of mcconnellite in the molten glass demonstrates its high thermal stability and excellent performance as a black ceramic pigment. Comparable results are obtained for samples glazed in a microwave kiln.

Using a microwave kiln, the blackness of the samples improves compared to electric firing. Among the powders, crednerite stands out with R_Vis_ = 4.85%, corresponding to 95.15% absorption of visible light. In contrast, for the glazed samples, although an improvement in blackness was observed in all cases (except for the mcconnellite sample) when using the microwave kiln, the optimal process remains glazing in the electric kiln. This is because glazing with the microwave kiln leads to numerous pinhole defects, likely associated with copper reduction promoted by microwave processing.

For samples glazed in an electric kiln, crednerite exhibited an R_Vis_ value of 5.10 (94.9% absorption), whereas mcconnellite showed the highest blackness, with R_Vis_ = 1.41 (98.59% absorption). Among the samples glazed in a microwave kiln, crednerite reached R_Vis_ = 4.27 (95.73% absorption), and mcconnellite achieved R_Vis_ = 2.63 (97.37% absorption), which was slightly inferior to the corresponding glazed in the electric furnace.

An increase in the electric firing temperature from 1000 to 1100 °C resulted in an enhancement of mcconnellite blackness. In contrast, increasing the microwave power from 800 W to 900 W produced very similar values (R_Vis_ ≈ 1.40%).

The thermal stability of mcconnellite’s blackness was confirmed at 1200 °C, together with its compatibility with porcelain glazes with high maturation temperature (1190 °C). The partial substitution of copper by iron and manganese further improved colour performance. The undoped mcconnellite powder maintained an intense black colour at 1200 °C (R_Vis_ = 8.89%), although this value was higher than those recorded for the doped composites (R_Vis_ = 3.27 and 3.23 for the Fe-doped and Fe–Mn-doped samples, respectively). The pigment also exhibited good resistance to high-maturation porcelain glazes (1190 °C), with R_Vis_ = 4.37, which was slightly inferior to the doped counterparts (R_Vis_ = 5.96 and 4.45 for the Fe- and Fe–Mn-doped samples, respectively), which displayed a brownish coloration attributed to the loss of the composite effect.

Among the delafossites investigated, only mcconnellite subjected to microwave-assisted firing (R_Vis_ = 1.40%, corresponding to 98.60% visible light absorption) emerges as a promising candidate for an ultra-black ceramic pigment (R_Vis_ < 1%), following further optimization under ZnO-free glaze conditions and a firing temperature of approximately 1000 °C. Considering the blackness in powder form, microwave-fired crednerite (R_Vis_ = 4.85; 95.15% absorption) and the Fe- and Fe–Mn-doped mcconnellite composites (R_Vis_ = 3.27 and 3.23%, respectively) appear as potential candidates for deep-black pigment applications (RVis < 3%), benefiting from the composite effect between the delafossite and chromium spinel phases. In addition, microwave-fired crednerite demonstrated promising deep-black behaviour in glazed samples (R_Vis_ = 4.27; 95.73% absorption).

## Figures and Tables

**Table 1:** Comparison of black ceramic pigments published in the recent literature.

		Powder		Glaze	
	Reference	L*a*b*	R_Vis_/R_NIR_/R (%)	L*a*b* wt%(T°C)	R_Vis_/R_NIR_/R (%)
Vantablack	[10]	0.2/0.9/−1	0.035/-/-	--	--
Carbon Black	[11]	20.2/0.1/0.1	3/3/3	--	--
Ferrochromite (Co,Ni)(Fe,Cr)_2_O_4_	[13,14]	41.0/1.0/1.0	3.5/-/-	33.0/1.0/0.05 5(1080)	4.5/-/-
CuCr_2_O_4_	[16]	40.5/0.1/−0.1	4.2/3.4/3.8	26.3/−0.3/−0.5 0.5(800)	3.8/4.5/4.1
CuCrO_2_	[17]	36.5/−4.8/−0.8	9.9/11.2/10.5	11.3/−0.6/−3.05 5(1000)	1.4/1.9/1.6
Mn,Ni-CuFe_5_O_8_	[18]	21.36/1.1/−0.24		16.95/−0.18/−0.12	
Fe_1.2_Cr_0.8_O_3_	[19]	42.3/−0.3/0.6	4/23/14	34.1/2.6/−3.75 5(1000)	4/17/10
YMnO_3_	[19]	27.7/−1.2/−3.5	3/39/19	31.9/1.6/1.15 5(1000)	7/31/18
Sr_4_CuMn_2_O_9_	[19]	43.9/7.2/6.3	7/51/29	57.9/2.8/0.25 5(1000)	25/41/33
Sr_2_(Mg_0.5_Mn_0.5_)Ge_2_O_7_	[19]	31.8/−0.1/−8.3	5/32/17	49.6/12.8/13.75 5(1000)	25/68/44

**Table 2:** Characterization of black samples: fired at 1100 °C for 3 h in an electric kiln (CE), fired at 900 W for 30 min in a microwaves kiln (MW). ΔE* is referred to as Carbon Black (L*a*b* = 20.2/0.1/0.1).

SAMPLE	L*a*b*	R_Vis_/R_NIR_/R (%)	E_g_ (eV)	ΔE*
POWDER				
1MnCE	27.32/1.18/0.40	5.47/6.06/5.73	1.31	7.2
2FeCE	31.14/−0.29/−2.47	6.87/7.10/6.98	1.42	11.2
3CrCE	28.66/−4.17/1.17	6.10/8.26/7.03	1.40	9.5
1MnMW	25.23/3.25/5.18	4.85/6.36/5.51	1.31	7.8
2FeMW	29.18/0.56/−1.70	6.45/13.40/9.52	1.47	9.2
3CrMW	31.05/−2.39/−0.15	6.98/7.73/7.31	1.40	11.1
**GLAZED ELECTRIC KILN** **5 wt% with glaze 1000**				
1MnCE	32.49/−1.93/−6.73	7.23/13.11/9.88	1.21	14.2
2FeCE	25.53/0.60/−5.93	5.02/13.99/9.01	1.21	8.1
3CrCE	24.70/−0.77/0.32	4.40/4.81/4.58	1.45	4.6
1MnMW	26.80/−0.63/−4.90	5.10/12.51/8.41	1.31	8.3
2FeMW	31.25/−0.12/−6.04	7.14/15.40/10.82	1.16	12.6
3CrMW	23.52/−0.02/−0.98	1.41/2.14/1.77	1.38	3.5
**GLAZED MICROWAVES** **5 wt% with glaze 1000**				
1MnCE	31.10/−0.77/1.77	6.37/8.50/7.32	1.50	11.1
2FeCE	28.60/−2.63/1.25	5.70/8.79/7.06	1.41	8.9
3CrCE	31.94/−4.67/3.88	5.95/9.42/7.50	1.80	13.2
1MnMW	24.64/0.02/0.40	4.27/5.93/5.01	1.51	4.5
2FeMW	34.30/−5.94/2.31	6.99/10.66/8.64	1.48	15.4
3CrMW	24.25/−1.41/−0.23	2.63/6.63/5.42	1.49	4.3

**Table 3:** XRD results. Crystalline phases: S (CuMn_2_O_4_), C (CuMnO_2_ Crednerite), D (CuFeO_2_ Delafossite), M (CuCrO_2_ mcconnellite), U (CuO), B (Mn_2_O_3_), and E (eskolaite Cr_2_O_3_), T (CuCr_2_O_4_). Intensity: vs (very strong), s (strong), m (medium), W (weak), and vw (very weak).

SAMPLE	Crystalline Phases
11MnCE	S (vs) C (m) U, B (vw)
2FeCE	D (vs) U, B (vw)
3CrCE	M (s) E, U (vw)
1MnMW	S (s) C (w) U, B (vw)
2FeMW	D (vs) U (s)
3CrMW	M (s) T (vw)

**Table 4:** Cell parameters of identified crystalline phases.

Sample	Identified Crystalline Phases		References
**CuMnO_2_-CE**	CuMn_2_O_4_ cubic (ICDD 74–2422) a = 8.308(1) Å	CuMnO_2_ monoclinic (ICDD 50-0860) β = 104.08° a = 5.677(1) Å b = 2.801(2) Å c = 6.063(2) Å V = 93.5(3) Å^3^	[34] CuMnO_2_ monoclinic (*C2/m* ICDD 50-0860) β = 104.03(3)° a = 5.592(3) Å b = 2.883(1) Å c= 5.892(3) Å V = 92.1(1) Å^3^ [35] β = 104.02(3)° a = 5.596(2) Å b = 2.880(1) Å c = 5.899(2) Å [38] CuMn_2_O_4_ cubic *Fd3m* ICDD 74-2422 a = 8.308 Å
**CuMnO_2_-MW**	CuMn_2_O_4_ cubic ICDD 74–2422) a= 8.308(1) Å	--	
**CuFeO_2_-CE**	CuFeO_2_ (ICDD-75-2146) a = b = 3.093(1) Å c = 17.109(1) Å V = 136.0(3) Å^3^	^--^	[39] (ICDD-75-2146) a = b = 3.04 Å c = 17.15 Å V = 136.8668 Å^3^
**CuFeO_2_-MW**	CuFeO_2_ (ICDD-75-2146) a = b = 3.002(1) Å c = 17.392(1) Å V = 135.8(3) Å^3^	^--^	
**CuCrO_2_-CE**	CuCrO_2_ (ICDD 39-0247) a = b = 2.968(5) Å c = 17.002(3) Å V= 129.7(4) Å^3^	^--^	[40,41] ICDD 39-0247 *a* = 2.9480(2) Å *c* = 17.033(2) Å V = 127.9731 Å^3^
**CuCrO_2_-MW**	CuCrO_2_ (ICDD 39-0247) a = b = 2.967(4) Å c = 17.021(2) Å V = 129.9(5) Å^3^	^--^	

**Table 5:** Comparison of mcconnellite obtained with literature results (reference [17]).

SAMPLE	L*a*b*	R_Vis_/R_NIR_/R (%)	E_g_ (eV)	ΔE*
**POWDER**				
*Electric kiln*				
3CrCE **1000 °C**/3 h	42.6/−5.4/0.1	13.8/14.1/13.9	1.26	23.1
3CrCE **1100 °C**/3 h	28.66/−4.17/1.17	6.10/8.26/7.03	1.40	9.5
*Microwave Kiln*				
3CrMW **800 W** 30 min	36.5/−4.8/−0.8	9.9/11.2/10.5	1.30	17
3CrMW **900 W** 30 min	31.05/−2.39/−0.15	6.98/7.73/7.31	1.40	11.1
**GLAZED ELECTRIC KILN** **5 wt% with glaze 1000**				
*Electric kiln*				
3CrCE **1000 °C**/3 h	26.9/−0.1/−2.3	5.3/5.0/5.2	1.0	7.2
3CrCE **1100 °C**/3 h	24.70/−0.77/0.32	4.40/4.81/4.58	1.45	4.6
*Microwave Kiln*				
3CrMW **800 W** 30 min	11.3/−0.6/−3.0	1.4/1.9/1.6	0.93	9.5
3CrMW **900 W** 30 min	23.52/−0.02/−0.98	1.41/2.14/1.77	1.38	3.5

## Data Availability

The original contributions presented in this study are included in the article. Further inquiries can be directed to the corresponding author.

## Abbreviations

SSA	Selective solar absorber
IR	Infrared
XRD	X-ray Diffraction
SEM	Scanning electron microscopy with energy-dispersive spectroscopy
ICDD	International Centre for Diffraction Data
UV-Vis-NIR	Ultraviolet-visible-near-infrared
